# Relationship Between High Glycated Hemoglobin and Severity of Coronary Artery Disease in Type II Diabetic Patients Hospitalized With Acute Coronary Syndrome

**DOI:** 10.7759/cureus.13734

**Published:** 2021-03-06

**Authors:** Fahad R Khan, Jabar Ali, Rizwan Ullah, Zair Hassan, Safi Khattak, Gul Lakhta, Nooh Gul

**Affiliations:** 1 Cardiology, Lady Reading Hospital, Peshawar, PAK; 2 Cardiology/ Interventional Cardiology, Lady Reading Hospital, Peshawar, PAK; 3 Gynecology and Obstetrics, Lady Reading Hospital, Peshawar, PAK

**Keywords:** coronary artery disease (cad), glycated hemoglobin (hba1c), acute coronary syndrome (acs), stemi, nste-acs, ste-acs, angiographic findings

## Abstract

Introduction

Diabetes mellitus (DM) is a chronic metabolic disease. It is the principal cause behind the high morbidity and mortality attributed to cardiovascular disease. This article’s objective was to determine a connection between high glycated haemoglobin levels (HbA1c) and coronary artery disease (CAD).

Materials and Methods

Cross-sectional research took place at the lady reading hospital, Peshawar, Pakistan, from 1st July 2020 to 31st December 2020. In this study, one hundred fifty-one type II diabetic patients took part. We labelled all of them as acute coronary syndrome (ACS) on arrival. Non-probability consecutive random sampling technique was used for sampling. We categorized patients based on their HbA1c levels into two groups. These groups included good glycemic control (HBA1c*≤*7. 5%) and patients with poor glycemic control (HBA1c *≥*7.5%). We classified the angiographic results of these patients as normal coronary arteries (NCAs), single vessel disease (SVD), double vessel disease (DVD), and triple vessel disease (TVD). Continuous variables such as age, weight, height, and body mass index (BMI) between HBA1c levels were analyzed using the Mann-Whitney U test. The fisher’s exact test was performed to compare the categorical variables between the two classes.

Results

Of the total 151 patients, 89 (58.9%) were males, and the rest were female. The mean age was 55.4 ± 11.2 years. The most common risk factors were diabetes and hypertension, whereas ST-segment elevation myocardial infarction (STEMI) was the most common presentation. 107 (70.86%) patients had poor glycemic control (HbA1c>7.5%). Coronary angiographies showed TVD in 77 (50.99%) patients. Among these patients with TVD, 6 (14%) patients had good glycemic control, while 71 (66%) patients had poor glycemic control, which is significant (P*≤*0. 001). None of the patients with poor glycemic control had NCAs.

Conclusion

This article found a link between high levels of HbA1c and the degree of coronary artery disease (CAD) among diabetic patients. Our study’s results demonstrated that high HbA1c was related to severe CAD. It would need additional studies with a large sample size to evaluate the more profound impact of HBA1c on coronary arteries.

## Introduction

Coronary artery disease (CAD) is the world’s number one cause of death over the past decade [1]. In 2013, approximately 7.5 million deaths globally were attributed to CAD [2]. The leading risk factor for CAD is diabetes mellitus (DM) [3-4]. Pakistan has 11.4% of diabetes in the age group 25 years and older [5]. A third of the deaths incurred by CAD are linked with diabetes. Patients with diabetes are at higher risk of developing CAD [6]. It is characterized by widespread coronary atherosclerotic disease. The cardiovascular system’s involvement in diabetes is largely the result of oxidative stress caused by inflammation, insulin resistance, hyperglycemia, and a range of other risk factors [7]. Patients with acute coronary syndrome (ACS) and concomitant diabetes mellitus (DM) have a two to four-fold increase in major cardiovascular adverse events (MACE) than non-diabetic patients. The increase is mainly attributed to hyperglycemic angiopathy [8].

Glycated haemoglobin (HbA1c) is a standard marker of long-term diabetic regulation in diabetic patients. Several studies have confirmed the association of HBA1c with cardiovascular diseases (CVD) [9-10]. It is universally recognized as an essential and more reliable measure of glycemic control than fasting glucose levels [4]. HbA1c levels are better than fasting glucose [11]. Patients with myocardial infarction (MI) and DM are vulnerable to multiple vessel involvement; the underlying mechanism remains subtle [12]. Already available studies have evaluated the relationship between HBA1c levels and the extent of CAD in diabetic patients with Non-ST elevation myocardial infarction (NSTEMI) or ST-elevation myocardial infarction (STEMI). However, none of the studies involved all three categories of ACS. Our research has fulfilled this gap. This study is the first study in Pakistan that has taken ACS into the limelight and incorporated patients from all three categories, i.e., patients diagnosed with unstable angina (UA), NSTEMI, and STEMI. It assessed the relationship between their HBA1c levels on admission and CAD severity on angiography.

## Materials and methods

We executed this cross-sectional, observational study in the Cardiac Care Unit (CCU) of the Cardiology department, Lady Reading Hospital, Peshawar, Pakistan, from 1st July 2020 to 31st December 2020. One hundred fifty-one diabetic patients labelled as ACS admitted via emergency, fulfilling the inclusion criteria, were enrolled in the study. All Patients presented to the Cardiac Care Unit (CCU) with chest pain. The technique used for sampling was non-probability consecutive random sampling.

After receiving the Ethical Review Committee’s approval and informed consent from the patients, they were included in the study based on inclusion criteria. After a detailed clinical evaluation, followed by an electrocardiogram, we classified patients into ST-elevation acute coronary syndrome (STE-ACS) and non-ST-elevation acute coronary syndrome (NSTE-ACS). ST-ACS included STEMI, whereas NSTE-ACS included patients with unstable angina (UA) and non-ST elevation myocardial infarction (NSTEMI) as per the guidelines issued by the America Heart Association for ACS in 2014 [13]. NSTEMI and UA differ in whether the severity of ischemia has caused the rise in cardiac troponin-I levels (a marker of myocardial injury) above the normal range, i.e. (between 0 and 0.4 ng/ml) or not. ECG in NSTE-ACS can show normal findings.

Inclusion Criteria

Participation in the study was voluntary, and only if patients fulfilled the inclusion criteria. Only diabetic patients were included in this study. Patients over 18 years of age were included in the study (this age has been chosen because most patients presenting to the Lady Reading Hospital with ACS come above this age). Presentation with ACS (as evidenced by signs and symptoms such as chest pain) was mandatory. We included patients with a new-onset ACS. Patients with both abnormal and normal ECG results were included. Abnormal ECG reports included changes in the ST-T segment, ST-elevations, T wave depressions, T wave inversions, biphasic T waves (initial positivity and terminal negativity), new-onset left bundle branch block (LBBB), right bundle branch block (RBBB), generalized ST depressions and elevation in ST-segment in AVR lead. We also added patients with normal ECG reports to the study. Normal ECG results mean lack of changes mentioned above in the ECG.

Exclusion Criteria

All those patients who had a history of ischemic heart disease (IHD) were excluded, including acute on chronic CAD. In this study, patients with cardiomyopathy and heart failure were exempted. Patients with a history of Chronic Kidney Disease (CKD), anaemia, and malignancy were also excluded from the study. This exclusion criterion ensured ACS was responsible for all ECG changes rather than confounding factors such as CKD, HF, valvular disease, and metabolic dysfunction.

Separate blood samples were collected from the patients to measure HbA1c levels. Based on their HBA1c, patients were categorized into two groups, i.e., patients with HbA1c less than 7.5%, i.e., good glycemic control, and patients with HbA1c over 7.5%, i.e., poor glycemic control. After coronary angiography, patients were classified based on the number of arteries involved, i.e., single-vessel disease (SVD), double vessel disease (DVD), and triple vessel disease (TVD) with greater than 70% stenosis. Those patients with no vessel involvement or lesion stenosis less than 70% were labelled as normal coronary arteries (NCAs).

Our data were not normally distributed; therefore, we carried out the Shapiro-Wilk test to determine data normality. Non-parametric tests were employed because of the skewness in the data. We presented continuous variables as mean ± standard deviation, while categorical variables as frequencies and percentages. A two-tailed Mann- Whitney U test was employed to analyze variation in continuous variables such as age, weight, height, and BMI amongst subgroups based on HBA1c levels. We compared categorical variables using the fisher’s exact test, taking p ≤0.05 as significant.

## Results

Out of the total 151 patients, 89 (58.9%) were male, and 62 (41.06%) were female. The mean age was 55.4 ± 11.2 years. Mean weight was 82.74± 16.1kg, while the mean and median body mass index (BMI) of the main group was 27.29 ± 5.37 and 23.18, respectively. For the subgroup of good glycemic control (HBA1c less than 7.5 %), BMI observations had an average of 27.49 (SD = 5.43, Mdn = 27.09). Whereas, for the subgroup with poor glycemic control (HBA1c more than 7.5%), the BMI observations had an average of 27.21 (SD = 5.37, Mdn = 23.18). The means HbA1c of the study sample was 10.2% ± 2.5%. Of the 151 diabetic patients, 22 (14.57%) had no other risk factors, 76 (50.3%) had associated hypertension. The remaining 53 (35.1%) patients had multiple risk factors, i.e., associated with hypertension, smoking, and a positive family history of premature CAD. We used the matching method to correct for the confounding risk factors. Based on their ECG findings and cardiac troponin-me (cTnI) levels, 107 (70.9%) patients were labelled as STEMI, 25 (16.6%) were NSTEMI, and 19 (12.6%) were Unstable Angina. Besides these findings, 44(29.1%) patients on arrival had good glycemic control, i.e., HBA1c less than 7.5%, whereas 107 (70.9%) had poor glycemic control, i.e., HBA1c over 7.5%. After carrying out coronary angiographies of these patients, only 9 (6%) patients had normal coronary arteries (NCA), 18 (11.9%) patients had SVD, DVD was present in 47 (31.1%) patients, whereas 77 (51.0%) patients had triple vessel disease (TVD). Baseline characteristics are listed in (Table 1).

**Table 1:** Baseline characteristics CAD- coronary artery disease; ACS- acute coronary syndrome; HBA1c- glycated hemoglobin; DM- diabetes mellitus; HTN- hypertension; EF- ejection fraction; NCAs- normal coronary arteries; SVD- single vessel disease; DVD- double vessel disease; TVD- triple vessel disease; STEMI- ST-elevation myocardial infarction; NSTEMI- NonST-elevation myocardial infarction; UA- unstable angina

Variable	Mean SD	N	%
Age	55.44 ± 11.27		
BMI	27.29 ± 5.37		
HBA1c Values	10.23 ± 2.58		
Weight	82.74 ± 16.11		
CAD			
Present		142	94.04
Absent		9	5.96
ACS			
STEMI		107	70.86
NSTEMI		25	16.56
UA		19	12.58
HbA1c levels			
Good glycemic control (HbA1c>6.4% and <7.5%)		44	29.14
Poor glycemic control (HbA1c>7.5% )		107	70.86
Risk factors			
DM		22	14.57
DM and HTN		76	50.33
DM, HTN, AND Family History of CAD		55	35.10
Coronary angiography findings			
NCAs		9	5.96
SVD		18	11.92
DVD		47	131.13
TVD		77	50.99
EF			
Normal EF( 50% to 70%)		24	15.89
Borderline (EF 41% to 49%)		33	21.85
Moderate reduced EF (31% to 40%)		62	41.06
Severely reduced EF less than or equal to 30%		32	21.1

The two-tailed Mann-Whitney U test was significant (p<.001) for age and insignificant for weight, height, and BMI. This finding suggested that the distribution of age for the group, good glycemic control (HbA1c<7.5%), differed significantly from the distribution of age in the group with poor glycemic control (HbA1c>7.5%) group. At the same time, this difference did not exist for weight, height, and BMI, as shown in (Table 2).

**Table 2:** Two-Tailed Mann-Whitney U test for age, weight, height and BMI by HbA1c HbA1c- Glycated haemoglobin levels; BMI- Body mass index

Variable	Mean Rank		U	Z	P
	Good glycemic control (HbA1c<7.5%)	Poor glycemic control (HbA1c>7.5%)			
Age	51.45	86.09	1274.00	-4.44	< .001
Weight	77.72	75.29	2429.50	-0.31	.755
Height	76.93	75.62	2395.00	-0.17	.861
BMI	77.84	75.24	2435.00	-0.33	.739

Categorical variables were compared between the two groups of HBA1c using the fisher’s exact test (Table 3).

**Table 3:** Comparison of categorical variables between groups using the fisher’s exact test DM- diabetes mellitus; HTN- hypertension; CAD- coronary artery disease

Variable	Good glycemic control (HbA1c>6.4% and <7.5%)	Poor glycemic control (HbA1c>7.5% )	P
Gender			< .001
Male	13 (30%)	76 (71%)	
Female	31 (70%)	31 (29%)	
Risk factors			.033
DM	6 (14%)	16 (15%)	.951
DM, HTN	29 (66%)	47 (44%)	.005
DM, HTN, smoking, and family history of CAD	9 (20%)	44 (41%)	.559
Duration of diabetes			.337
Less than 10 years	98	64.90	
more than 10 years	53	35.10	

Similarly, the severity of CAD in the form of the number of arteries involved was compared between subgroups of HBA1c, i.e., between good glycemic control and Poor glycemic control (Table 4).

**Table 4:** Coronary angiographic findings compared among HbA1c groups NCAs- Normal coronary arteries; SVD- Single vessel disease; DVD- Double vessel disease; TVD- Triple vessel disease

Variable	Good glycemic control (HbA1c<7.5%)	Poor glycemic control (HbA1c>7.5% )	P
NCAs	9 (20%)	0 (0%)	< .001
SVD	16 (36%)	2 (2%)	< .001
DVD	13 (30%)	34 (32%)	.788
TVD	6 (14%)	71 (66%)	< .001

## Discussion

In the present article, we have analyzed the link between glycated haemoglobin (HbA1c) on admission and severity of coronary artery disease in terms of the number of coronary arteries involved in patients presented with ACS to one of the largest hospitals in Pakistan. We found that HbA1c has a strong association with the severity of CAD in the form of an increased number of coronary arteries involved. As far as we know, none of the studies before has established this relationship. The majority of study participants were elderly and overweight.

The current study’s mean age was 55.00, with 90% of patients aged seventy and above. This study’s mean age was lower than the mean age of previous research findings by Cai and Ikeda et al. [14-15]. This statement signifies that diabetic patients are at risk of developing CAD at an early age in this part of the world.

Most of the participants were male in this study, which is consistent with the study conducted by Beig et al. [16]. Similarly, most of the patients were overweight. The male to female ratio to be overweight was 1:1, unlike previous studies, where females were more prone to obesity because of their sedentary lifestyle [17-18]. A literature search showed that data on the relationship between high HBA1c levels on arrival and the magnitude of CAD in diabetic patients with ACS is scarce [19].

Evidence supports the fact that developing countries like Pakistan lack knowledge and facilities for early patient rehabilitation, such as healthy chronic angina. Therefore, ACS is perhaps the most typical CAD presentation in developing countries than in developed countries [20]. STEMI has been the most frequent manifestation of ACS. Similarly, STEMI was the most common presentation in the present study, aligning with Dar et al. [21]. In this study, the most common angiographic finding in patients with HBA1c more than 7.5% were TVD. This finding is on par with the study results from Hegde SS et al. [22]. About 1/3 of the patients had a DVD, and only around 12% had SVD. These findings were contrary to earlier studies by Mishra and Patil et al., in which single-vessel involvement was predominant [23-24].

Conway et al. followed the Pittsburgh Epidemiology of Diabetes Complications cohort study for cardiovascular events, including fatal and nonfatal CAD. Baseline HbA1c was an independent risk factor for CAD in this cohort study [25]. Our research also supported Conway et al. study. In another study conducted by Karakoyun et al., an association between HBA1c and Syntax score II was established. They found out that raised HbA1c is linked to high syntax score in patients with DM, which favours our study finding thatHbA1c is associated with the severity of CAD [26].

Jibran et al. conducted a study on 100 patients. Among them, 70% were males, and 68% were diagnosed with diabetes; their mean age was 56.04 ± 9.24 years. Their mean HbA1c level was 7.0±1.3%. The purpose of their research was to determine the effect of rising HbA1c levels on the angiographic severity of CAD in diabetic patients diagnosed with NSTEMI. The association between increasing HbA1c levels and angiographic severity of CAD was significant [27]. These findings are almost similar to our finding’s, i.e., the mean age of our study sample was 55.44 ± 11.27 years, and the mean HBA1c was 10.23 ± 2.58%. Furthermore, we also included patients presented with STEMI and UA besides NSTEMI. Therefore, our study had younger patients with poor glycemic control compared to Jibran MS et al.

Eeg-Olofsson et al. did a study on 7,454 patients, ages ranged from 20 to 60 years, and DM duration was 1 to 35 years. They found that the risk of CAD continued to rise with higher levels of HbA1c, independent of conventional risk factors [28]. This research article’s findings are identical to the study of Eeg-Olofsson et al. Still, patients in our sample were elderly, and 65% had a diabetes duration of fewer than ten years.

Kamal et al. conducted a study on 150 patients who underwent coronary angiography, and their Gensini score (GS) was measured. Their study included both diabetic and non-diabetic. Among them, 64.7% had diabetes mellitus, and 54.7% were hypertensive. 74.23% of the diabetic group patients had HbA1c higher than or equal to 7%, with a mean HbA1c of 9.7 ± 2.2. The means GS was 41 ± 31.3. there was a significant positive correlation between HbA1c and fasting plasma glucose levels. Our study’s sample size and the mean HBA1c level were more or less the same as that of Kamal et al. Though our study did not consider the Gensini score (GS), it backed the results by Kamal et al.’s research [29].

In another study by Sahal et al., who enrolled 905 patients with CAD, their results exhibited a positive relationship between raised HbA1c levels in people with diabetes and a higher syntax score (≥23) than non-diabetics [30]. Even though our study did not include syntax scoring, yet the findings of our research-backed this study.

HBA1c’s importance in assessing the magnitude of CAD is demonstrated by this study’s findings and previous studies’ results. The current study result showed that more coronary arteries were involved in patients with higher HBA1c on arrival. It strengthens the well-non fact that HbA1c is an independent risk factor for the severity of CAD. While encountering diabetic patients with concomitant CAD, this study, along with other similar studies, will encourage healthcare professionals to make prudent decisions in patients with inadequate glycemic control on arrival.

Limitations

Our study has a few limitations. The study duration was short, and no clinical follow-up was carried out. Besides, the study sample was too small, and it was a one centre study. We did not consider a scoring system for measuring the severity of CAD, i.e., Gensini score or syntax score in our study. Patients’ fasting and postprandial glucose testing were not considered, which is another notable limitation. We did not consider the patients’ physical activity, which has a prominent effect on CAD. Due to some unavoidable circumstances, unfortunately, we did not include the results of renal function tests in our study, and we did not carry out urine albumin measurement. It may require a multicenter study with a larger sample size to better estimate study parameters. Further studies are needed on a large scale, including trials and longitudinal studies adjusted for confounding variables to use HBA1c as a predictor of the severity of CAD and fulfil the current research gaps.

## Conclusions

Our research concluded that high HbA1C is coupled with increased severity of coronary artery disease in diabetic patients presenting with ACS; the higher the HbA1c level, the more severe is the coronary artery disease. This study will help clinicians choose between high-risk and low-risk patients using HBA1c as a crude marker of disease severity, keeping into consideration those conditions in which HBA1c is falsely elevated or decreased, e.g., anaemia, hemoglobinopathies and chronic kidney disease (CKD). More research is needed to understand the association between HBA1c and CAD, and the answer lies in further extensive randomized studies.

## References

[1] (2017). Classification and Diagnosis of Diabetes.

[2] Wang H, Naghavi M, Allen C (2016). Global, regional, and national life expectancy, all-cause mortality, and cause-specific mortality for 249 causes of death, 1980-2015: a systematic analysis of the global burden of disease study 2015. Lancet.

[3] Cicek G, Uyarel H, Ergelen M (2011). Hemoglobin A1c as a prognostic marker in patients undergoing primary angioplasty for acute myocardial infarction. Lipids Health Dis.

[4] Hong L-F, Li X-L, Guo Y-L (2014). Glycosylated hemoglobin A1c as a marker predicting the severity of coronary artery disease and early outcome in patients with stable angina. Coron Artery Dis.

[5] Tomizawa N, Nojo T, Inoh S, Nakamura S (2015). Difference of coronary artery disease severity, extent and plaque characteristics between patients with hypertension, diabetes mellitus or dyslipidemia. Int J Cardiovasc Imaging.

[6] Pradhan AD, Rifai N, Buring JE, Ridker PM (2007). Hemoglobin A1c predicts diabetes but not cardiovascular disease in non-diabetic women. Am J Med.

[7] Song P, Xu J, Song Y, Jiang S, Yuan H, Zhang X (2015). Association of plasma myeloperoxidase level with risk of coronary artery disease in patients with type 2 diabetes. Dis Markers.

[8] Pai JK, Cahill LE, Hu FB, Rexrode KM, Manson JE, Rimm EB (2013). Hemoglobin A1c is associated with increased risk of incident coronary heart disease among apparently healthy, non-diabetic men and women. J Am Heart Assoc.

[9] Selvin E, Steffes MW, Zhu H (2010). Glycated hemoglobin, diabetes, and cardiovascular risk in non-diabetic adults. N Engl J Med.

[10] Arnold LW, Hoy WE, Sharma SK, Wang Z (2016). The association between HbA1c and cardiovascular disease markers in a remote indigenous Australian community with and without diagnosed diabetes. J Diabetes Res.

[11] Guo F, Moellering DR, Garvey WT (2014). Use of HbA1c for diagnoses of diabetes and prediabetes: comparison with diagnoses based on fasting and 2-hr glucose values and effects of gender, race, and age. Metab Syndr Relat Disord.

[12] Olsson M, Schnecke V, Cabrera C, Skrtic S, Lind M (2015). Contemporary risk estimates of three HbA1c variables for myocardial infarction in 101,799 patients following diagnosis of type 2 diabetes. Diabetes care.

[13] Amsterdam EA, Wenger NK (2015). The 2014 American College of Cardiology ACC/American Heart Association guideline for the management of patients with non-ST‐elevation acute coronary syndromes: ten contemporary recommendations to aid. Clin Cardiol.

[14] Cai A, Li G, Chen J (2014). Glycated hemoglobin level is significantly associated with the severity of coronary artery disease in non-diabetic adults. Lipids Health Dis.

[15] Ikeda F, Doi Y, Ninomiya T (2013). Hemoglobin A1c even within non-diabetic level is a predictor of cardiovascular disease in a general Japanese population: the Hisayama study. Cardiovasc Diabetol.

[16] Beig JR, Shah TR, Hafeez I (2017). Clinico-angiographic profile and procedural outcomes in patients undergoing percutaneous coronary interventions: the Srinagar registry. Indian Heart J.

[17] Bhatt P, Parikh P, Patel A (2015). Unique aspects of coronary artery disease in Indian women. Cardiovasc Drugs Ther.

[18] Krishnan M, Zachariah G, Venugopal K (2016). Prevalence of coronary artery disease and its risk factors in Kerala, South India: a community-based cross-sectional study. BMC Cardiovasc Disord.

[19] Dutta B, Neginhal M, Iqbal F (2016). Glycated hemoglobin (HbA1c) correlation with severity of coronary artery disease in non-diabetic patients-a hospital based study from North-Eastern India. J Clin Diagnostic Res.

[20] Sharma L, Hussain ME, Verma S (2017). Effect of recovery modalities on blood lactate clearance. Saudi J Sports Med.

[21] Dar MI, Beig JR, Jan I, Shah TR, Ali M, Rather HA, Tramboo NA (2020). Prevalence of type 2 diabetes mellitus and association of HbA1c with severity of coronary artery disease in patients presenting as non-diabetic acute coronary syndrome. Egypt Heart J.

[22] Hegde SS, Mallesh P, Yeli S (2014). Comparitive angiographic profile in diabetic and non-diabetic patients with acute coronary syndrome. J Clin Diagn Res.

[23] Mishra TK, Das B (2016). ST-segment elevated acute myocardial infarction: changing profile over last 24 years. J Assoc Physicians India.

[24] Patil VC, Patil S, Sabale S, Agrawal V, Mhaskar D (2015). Study of percutaneous coronary intervention in patient with coronary artery disease at tertiary care teaching hospital. JKIMSU.

[25] Conway B, Costacou T, Orchard T (2009). Is glycaemia or insulin dose the stronger risk factor for coronary artery disease in type 1 diabetes?. Diab Vasc Dis Res.

[26] Karakoyun S, Gökdeniz T, Gürsoy MO (2016). Increased glycated hemoglobin level is associated with SYNTAX score II in patients with type 2 diabetes mellitus. Angiology.

[27] Jibran MS, Khan SB, Habib SA (2018). Shawana : Relationship of glycated hemoglobin with severity of coronary artery disease in patients with non-ST elevation myocardial infarction. Pak Heart J.

[28] Eeg‐Olofsson K, Cederholm J, Nilsson PM, Zethelius B, Svensson AM, Gudbjörnsdóttir S, Eliasson B (2010). New aspects of HbA1c, as a risk factor for cardiovascular diseases in type 2 diabetes: an observational study from the Swedish national diabetes register. J Intern Med.

[29] Kamal AM, Mostafa AA, Bayoumi BM (2019). Severity of atherosclerotic coronary artery disease in relation to glycated hemoglobin level in diabetic patients. Menoufia Med J.

[30] Sahal N (2019). Impact of glycated hemoglobin level on severity of coronary artery disease in non-diabetic patients. J Cardiol Curr Res.

